# Residual lymph node status is an independent prognostic factor in esophageal squamous cell Carcinoma with pathologic T0 after preoperative radiotherapy

**DOI:** 10.1186/s13014-015-0450-4

**Published:** 2015-07-11

**Authors:** Qifeng Wang, Shufei Yu, Zefen Xiao, Xiao Liu, Wencheng Zhang, Xun Zhang, Jie He, Kelin Sun, Ting Xu, Qinfu Feng, Zongmei Zhou, Lvhua Wang, Weibo Yin

**Affiliations:** Department of Radiation Oncology, Cancer Institute and Hospital, Chinese Academy of Medical Sciences, Beijing, 100021 People’s Republic of China; Department of Radiation Oncology, Sichuan Cancer Hospital, Chengdu, 610041 People’s Republic of China; Department of Pathology, Cancer Institute and Hospital, Chinese Academy of Medical Sciences, Beijing, 100021 People’s Republic of China; Department of Thoracic Surgery, Cancer Institute and Hospital, Chinese Academy of Medical Sciences, Beijing, 100021 People’s Republic of China; Department of Radiation Oncology, MD Anderson Cancer Center, University of Texas, Houston, Texas USA

**Keywords:** Esophageal squamous cell carcinoma (ESCC), Preoperative radiotherapy, Lymph node metastases

## Abstract

**Objective:**

To evaluate the prognostic factors affecting survival in esophageal squamous cell Carcinoma (ESCC) patients with pathologic T0 (ypT0) underwent preoperative radiotherapy.

**Patients and methods:**

Two hundred and ninety-six patients with ESCC who had received preoperative radiotherapy from 1980 to 2007 were retrospectively analyzed. One hundred patients were ypT0 after preoperative radiotherapy. Univariate and multivariate analyses were performed to evaluate the predictive impact of residual lymph node status on overall survival (OS) and progression-free survival (PFS).

**Results:**

Among the originally analyzed 296 patients, 100 (33.7 %) patients had ypT0, including 78 patients (78 %) with ypT0N0, and 22 patients (22 %) with ypT0N1. The 5-year OS of the total patients was 42.4 %. Patients with ypT0N0 have significant improved 5-year OS and PFS than ypT0N1 patients (OS: 50.7 % vs 13.6 %, *P* = 0.004; PFS: 49.6 % vs 13.6 %, *P* = 0.003). In multivariate analysis, residual lymph node status was also an independent prognostic factors for OS (HR: 0.406, 95 % CI: 0.240–0.686, *P* = 0.001) and PFS (HR: 0.427, 95 % CI: 0.248–0.734, *P* = 0.002).

**Conclusion:**

Our results indicate that patients with ypT0N0 after preoperative radiotherapy had significantly better OS and PFS than patients with ypT0N1 in ESCC. Residual nodal metastasis of ESCC patients with pathological complete response of the primary tumor after neoadjuvant radiotherapy does influence prognosis.

## Background

Outcomes for patients with cancer of the esophagus or gastroesophageal junction (GEJ) remain poor with surgery alone. A variety of adjuvant treatments have been studied in an attempt to improve survival. Recently, a lot of attention has been focused on the neoadjuvant radiotherapy/chemoradiotherapy (RT/CRT). Preoperative multimodality therapy could improve the rate of tumor resection, reduce the lymph nodes metastasis, decrease the rate of local recurrence, and also improve survival. Randomized and non-randomized [1–10] trials have shown about 30 % of patients who underwent preoperative RT or CRT had experienced pathologic complete response (pCR) in the resected specimen [1–13].

A previous study showed that when reclassified patient stage according to the AJCC 7th edition TNM criteria after neoadjuvant CRT, the 5-year overall survival (OS) of patients with ypT0N1 was significantly lower than ypT0N0 patients, and similar to pathologic partial response (pPR) stage II patients [14]. In addition, Rizk *et al.* [6] reported that residual nodal disease was the most important prognostic factor for the patients with adenocarcinoma cancer of the esophagus undergoing CRT followed by surgery. However one study from Korea reported that residual nodal metastasis was not a significant prognostic factor for OS and PFS in ypT0-1 ESCC patients underwent preoperative CRT. So the role of residual lymph node status in predicting prognosis still needs to be further studied.

Our aim of this study was to evaluate the predictive effect of residual nodal metastasis for OS and progression-free survival (PFS) in ESCC patients after neoadjuvant therapy.

## Materials and methods

### Patients

A total of 296 patients with ESCC who had undergone neoadjuvant radiotherapy followed by surgery in our hospital between January 1980 and November 2007 were retrospectively analyzed. Before treatment start, all patients underwent a barium swallow, upper gastrointestinal endoscopy, B-type ultrasonography of the neck and abdomen, collection of blood parameters (including hematology), and biochemical investigations (including liver function tests). The inclusion criteria included 1) Karnofsky performance score ≥80, 2) tumors ≤12 cm in length on endoscopy and/or barium swallow, 3) the capability to take semifluid food. The exclusion criteria were 1) hoarseness of the voice, 2) have active bleeding, 3) perforation of the esophagus, 4) have remote metastasis, 5) prior malignancy history. The cancers in the lower esophageal sphincter were all identified near the opening of the sphincter, and no clear invasion into the stomach was observed. Finally, 100 (33.7 %) patients with primary tumor pCR were enrolled into this study. The Academic Committee of Chinese Academy of Medical Sciences approved this study.

### Radiotherapy

The external beam radiotherapy was performed with an 8-MV linear accelerator for the whole 296 patients. Anterior-posterior-opposed radiation fields (the whole mediastinum and the left gastroepiploic lymphatic chain) were used in 284 patients (95.5 %) for conventional radiotherapy. Intensity-modulated radiotherapy (IMRT) and three-dimensional conformal radiotherapy (3D-CRT) was applied in 12 patients (4.5 %). For the 100 patients with pCR, 95 patients underwent conventional radiotherapy and 5 patients received 3D-CRT or IMRT.

Gross tumor volume (GTV) defined by a 0.5 cm margin in the lateral and anterior-posterior directions of the CT scan. The clinical target volume (CTV) in this study was re-created using a 3 cm margin in the proximal and distal direction (following the course of the esophagus) beyond the barium esophagram, endoscopic examination. Finally, the planning treatment volume (PTV) was defined by including additional 1-cm proximal and distal margins and 0.5 cm radial margin based on the CTV. 95 % of the PTV received 40–44 Gray (Gy) of the prescribed dose with 2 Gy/fraction/day and 5 days per week. The median dose of radiotherapy was 40Gy. Two hundred and seventy (91.5 %) patients received 40Gy and 25 patients (8.5 %) received 42-50Gy.

### Surgery

Surgery was carried out after median 4 weeks (2–8 weeks) rest after neoadjuvant RT. Two hundred and forty patients (81.1 %) had undergone R0 resection and 56 patients (18.9 %) had R2 resection. Two-field lymph node dissection was routinely performed, and three-field lymph node dissection was performed for patients with suspected or biopsy proved metastases in the cervical or supraclavicular lymph nodes. A total of 3577 lymph nodes were removed for all 296 patients, the median was 11 for each patient (ranging: 1–53). For the 100 patients with ypT0, 1119 lymph nodes were removed in total and the median was 10 for each patient (ranging: 1–46).

After surgery, follow-up included a visit to the esophageal cancer clinic every 3–6 months for the first 2 years and every 6–12 months thereafter. CT scans and esophagogastroscopy assessments were performed every 12 months for the first 5 years and whenever clinically indicated. The median duration of follow-up was 25 months (range: 6–250 months).

### Pathological characteristics

A group of pathologists examined the entire specimen with primary and dissected lymph nodes. Based on the presence or absence of viable tumor at the primary site, the specimens were divided into tumor pCR or residual disease. The tumors were classified as pCR when no cancer was found at the primary tumors, such that the following conditions existed: there were no residual tumors in general, the neoplastic cells had completely disappeared, and/or there was fibrosis in the tumor bed, reduction in vascularity, chronic inflammatory cell infiltration and scar formation were observed by microscopy.

### Statistical analysis

All analyses were performed using SPSS 13.0 (SPSS Inc.). The categorical variables between groups were compared using Pearson’s Chi square test. OS was calculated from the date of surgery to the date of death or the last contact date. PFS was calculated from the date of surgery to the date of the first observation of recurrence, date of death or last follow-up without recurrence. The survival was assessed using the Kaplan-Meier curve and log-rank test. Cox proportional model was used for multivariate analysis. All statistical tests were two-sided with significance defined as *P* <0.05.

## Results

### Patients

The clinical characteristics of the study patients are shown in Table 1. For 100 patients with ypT0, the median age was 55 years (27–78 years). Seventy-eight patients (78.0 %) were ypT0N0, and 22 patients (22.0 %) were ypT0N1 after neoadjuvant RT. No significant differences existed in clinical characteristics between the two groups of patients. Among the 22 patients with ypT0N1, 3 patients had positive node in paracardial and 8 patients had positive node in left gastric artery (1 have positive node in both section). A total of 67 positive nodes were dissected for these 22 ypT0N1 patients. 41, 8 and 18 positive nodes were found to metastasis in mediastinum, pericardial and left gastric artery respectively. Complications within 90 days post operation includes pneumonia (11 patients), anastomotic site leakage (5 patients), and anastomotic site stricture (6 patients). Postoperative 30-day mortality rate was 3.4 % (n = 10). Causality of death includes respiratory failure (6 patients) and myocardial infarction (4 patients).

**Table 1:** Characteristics of esophageal cancer patients grouped by pathologic N status in patients with pathologic complete response of primary tumors

Characteristic	Total n = 100	ypT0N0 n = 78	ypT0N1 n = 22	*P*-value
Gender				0.973
Male(ref)	77 (77.0)	60 (76.9)	17 (77.3)	
Female	23 (23.0)	18 (23.1)	5 (22.3)	
Age(year				0.746
<65	89 (89.0)	69 (88.5)	20 (90.9)	
≥65	11 (11.0)	9 (11.5)	2 (9.1)	
The length(cm)				0.321
<6	41 (41.0)	34 (43.6)	7 (31.8)	
≥6	59 (59.0)	44 (56.4)	15 (68.2)	
The location				0.323
Upper	34 (26.0)	23 (29.5)	3 (13.6)	
Middle	63 (63.0)	47 (60.3)	16 (72.7)	
Lower	11 (11.0)	8 (10.3)	3 (13.6)	
Clinical T stage^a^				0.729
T2	34 (34.0)	25 (32.1)	9 (40.9)	
T3	52 (52.0)	42 (53.8)	10 (45.5)	
T4a	14 (14.0)	11 (14.1)	3 (13.6)	
Clinical N stage^a^				0.461
N0(ref)	70 (70.0)	56 (71.8)	14 (63.6)	
N1	30 (30.0)	22 (28.2)	8 (36.4)	
Clinical stage^a^				0.791
Stage II	66 (66.0)	52 (66.7)	14 (63.6)	
Stage III	34 (34.0)	26 (33.3)	8 (36.4)	
Surgery				0.521
Lvor-Lowis ooproperation	64 (64.0)	48 (61.5)	16 (72.7)	
Mckeown	36 (36.0)	30 (38.5)	6 (27.3)	
Local recurrences				0.418
yes	17 (17.0)	12 (12.0)	5 (22.7)	
no	83 (83.0)	66 (66.0)	17 (77.3)	

Data are expressed as n (%) unless otherwise specified

AJCC American Joint Committee on Cancer, LNs lymph nodes, SD standard deviation

^a^ AJCC 2002 staging system

### 5-year OS and PFS

**Fig. 1:**
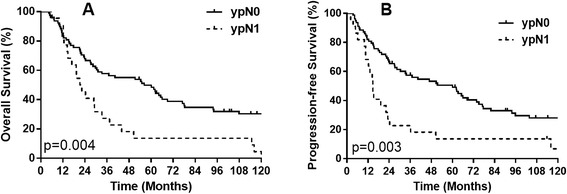
**a** overall survival (OS) curves of patients with primary tumor pCR. **b** Progression-free survival (PFS) curves of patients with primary tumor pCR. Patients with ypT0N1 disease have a significantly lower OS and PFS ((*P* = 0.004 and *P* = 0.003 respectively)

The 5-year OS was 42.4 % for ypT0 patients, 50.7 % for the ypT0N0 group and 13.6 % for the ypT0N1 group. The 5-year PFS was 41.2 % for ypT0 patients, 49.1 % for the ypT0N0 group and 13.6 % for the ypT0N1 group. Patients with N0 status after neoadjuvant radiotherapy have significant improved 5-year OS and PFS than N1 patients (*P* = 0.004 and *P* = 0.003 respectively, Fig. 1a-b).

### Univariate and multivariate analysis on overall survival and progression-free survival

Univariate analyses showed that female, clinical T2 stage, clinical TNM stage II, ypN0 status improved OS and PFS significantly (*P* = 0.017, 0.050, 0.032, 0.004 for OS, *P* = 0.038, 0.006, 0.063, 0.008 for PFS, data not shown). However, age, tumor location, tumor length, clinical N stage, and the operation style have no impact on OS or PFS in this study (*P* > 0.05). In multivariate analysis, ypN Status was also an independent prognostic factors for OS (HR: 0.406, 95 % CI: 0.240–0.686, *P* = 0.001) and PFS (HR: 0.427, 95 % CI: 0.248–0.734, *P* = 0.002) after adjustment for gender, age, clinical T stage and TNM stage (Table 2).

**Table 2:** Multivariate analysis of overall survival and progression-free survival in relation to clinical-pathologic characteristics

Characteristics	PFS		OS	
	HR (95 % CI)	*P* value	HR (95 % CI)	*P* value
Gender (Female vs Male)	1.936 (1.044–3.590)	0.036	2.039 (1.178–3.531)	0.011
Age (≥65 vs <65)	0.381 (0.175–0.831)	0.015	0.481 (0.236–0.980)	0.044
Clinical T stage (T3-4 vs T2)	0.928 (0.507–1.697)	0.808	1.010 (0.575–1.776)	0.972
Clinical TNM stage (III vs II)	0.697 (0.389–1.249)	0.226	0.692 (0.404–1.185)	0.180
ypN stage (N1vs N0)	0.427 (0.248–0.734)	0.002	0.406 (0.240–0.686)	0.001

Note: HR: hazard ratio, CI: confidence interval, ypN: post-therapy pathologic lymph node status; pCR: pathologic complete response; ypT0N1: pathologic complete response in tumor but residual lymph node metastasis after neoadjuvant radiotherapy and surgery

## Discussion

Our results showed that OS and PFS of patients with ypT0N1 after neoadjuvant RT were significantly worse than those of patients with ypT0N0, which indicated that residual nodal metastases after neoadjuvant RT does influence the prognosis of patients with pCR of the primary tumor underwent preoperative RT plus surgery. Several randomized clinical trials showed that pCR after neoadjuvant therapy plus surgery ranged from 11 to 43 % for esophageal carcinoma [1–13, 15–17]. pCR after CRT is most commonly used to predict survival for these patients [6, 18–20]. However, published studies are still insufficient for the prediction of survival in patients with primary tumor pCR [6, 19].

Rizk *et al.* [21] reported that residual nodal disease after CRT was the most important prognostic factor for the patients with adenocarcinoma of the esophagus undergoing CRT followed by surgery. In their study, the primary tumor pCR was 52.7 % (48/91) and the prevalence of ypT0N1 was 9.9 % (9/91) for ESCC (n = 91). Similar results were also reported by other studies. Kim *et al.* [14] reported primary tumor pCR was 64.6 % (29/45) and the prevalence of ypT0N1 was 8.9 % (4/45) in a subgroup analysis of ESCC (n = 45). However, Cho’s study showed residual lymph node metastases did not significantly influence prognosis in primary tumor pCR patients in ERCC [22].

In our study, the primary tumor pCR rate was 33.7 % (100/296) and the prevalence of ypT0N1 was 7.4 % (22/296) in ESCC. For ypT0N1 patients, the number of lymph node metastases of 1–3 and ≥4 were 17/100 and 5/100 respectively. The 5-year OS for patients with N, 1–3 N+ and ≥4 N+ were 49.8 %, 17.6 % and 0 respectively (P = 0.0002). In the current study, found lower OS in patients with higher number of lymph node metastases, but its predictive role for prognosis needs to be further investigated with larger samples.

The previous Korean study showed that when reclassified according to the AJCC 7th edition TNM criteria, the 5-year survival of patients with ypT0N1 was similar to pPR stage II patients [14]. Although the 7th edition AJCC/UICC [23] modified the TNM staging of esophageal carcinoma considering the number of lymph node metastases in 2009, there is still no conclusion as to whether the 7th TNM staging criteria could be used for lymph node metastases staging in ypT0 patients after preoperative RT [24]. Thus we recommend that future revisions may take consideration of patients with pathologic ypT0N1 for esophageal cancer.

In this study, the clinical target volume for radiation therapy included areas at risk for microscopic disease and lesions suspected on PET or CT scan. However, besides mediastinum (41 positive), the residual nodal metastasis was very common in abdominal lymph nodes (18 positive), especially in the left gastric arterial lymph nodes (18 positive). This indicated that a careful and complete dissection of abdominal lymph nodes is important after neoadjuvant therapy.

This study still has several limitations. First, this is a retrospective analysis with limited number of patients; selection bias may exist in unmeasured baseline characteristics, such as nutrition status, which may also be a source of confounding. Second, CT scan hasn’t been used in our institution before 1993 and endoscopic ultrasound hasn’t been used before 2000, so the clinical TNM depended on other exams such as barium esophagram, endoscopic examination, B-type abdominal ultrasound, and so on. Third, in this study only 4.5 % of patients underwent 3D-CRT or IMRT.

In conclusion, our study indicated that the status of the residual lymph nodes was significant factor for OS and PFS in ESCC patients with primary tumor pCR after preoperative radiotherapy. It is important to achieve a complete surgical resection of the primary tumor site and lymph nodes even in cases where neoadjuvant therapy is implemented

### Permissions

Permissions are unnecessary because all content herein was generated by the authors themselves.
